# Comparative Epidemiology of Highly Pathogenic Avian Influenza Virus H5N1 and H5N6 in Vietnamese Live Bird Markets: Spatiotemporal Patterns of Distribution and Risk Factors

**DOI:** 10.3389/fvets.2018.00051

**Published:** 2018-04-05

**Authors:** Kate C. Mellor, Anne Meyer, Doaa A. Elkholly, Guillaume Fournié, Pham T. Long, Ken Inui, Pawin Padungtod, Marius Gilbert, Scott H. Newman, Timothée Vergne, Dirk U. Pfeiffer, Kim B. Stevens

**Affiliations:** ^1^Veterinary Epidemiology, Economics and Public Health Group, Department of Pathobiology and Population Sciences, Royal Veterinary College, Hatfield, United Kingdom; ^2^Department of Animal Health, Ministry of Agriculture and Rural Development, Hanoi, Vietnam; ^3^Country Office for Vietnam, Food and Agriculture Organization of the United Nations, Hanoi, Vietnam; ^4^Spatial Epidemiology Laboratory, Université Libre de Bruxelles, Brussels, Belgium; ^5^Country Office for Ethiopia, Food and Agriculture Organization of the United Nations, Addis Ababa, Ethiopia; ^6^Maladies Infectieuses et Vecteurs Ecologie, Génétique, Evolution et Contrôle (MIVEGEC), Institut de Recherche pour le Développement (IRD), Montpellier, France; ^7^UMR 1225 INRA, ENVT Interactions Hôtes – Agents Pathogènes (IHAP), University of Toulouse, Toulouse, France; ^8^College of Veterinary Medicine & Life Sciences, City University of Hong Kong, Kowloon, Hong Kong

**Keywords:** avian influenza, epidemiology, live bird markets, poultry, spatial modelling, Vietnam, emerging infectious disease

## Abstract

Highly pathogenic avian influenza (HPAI) H5N1 virus has been circulating in Vietnam since 2003, whilst outbreaks of HPAI H5N6 virus are more recent, having only been reported since 2014. Although the spatial distribution of H5N1 outbreaks and risk factors for virus occurrence has been extensively studied, there have been no comparative studies for H5N6. Data collected through active surveillance of Vietnamese live bird markets (LBMs) between 2011 and 2015 were used to explore and compare the spatiotemporal distributions of H5N1- and H5N6-positive LBMs. Conditional autoregressive models were developed to quantify spatiotemporal associations between agroecological factors and the two HPAI strains using the same set of predictor variables. Unlike H5N1, which exhibited a strong north–south divide, with repeated occurrence in the extreme south of a cluster of high-risk provinces, H5N6 was homogeneously distributed throughout Vietnam. Similarly, different agroecological factors were associated with each strain. Sample collection in the months of January and February and higher average maximum temperature were associated with higher likelihood of H5N1-positive market-day status. The likelihood of market days being positive for H5N6 increased with decreased river density, and with successive Rounds of data collection. This study highlights marked differences in spatial patterns and risk factors for H5N1 and H5N6 in Vietnam, suggesting the need for tailored surveillance and control approaches.

## Introduction

Highly pathogenic avian influenza (HPAI) H5N1 virus (hereafter H5N1) is endemic to multiple Asian countries, including Vietnam, where the first recorded H5N1 outbreak occurred in 2003. Since then, costly control measures have been introduced, including culling of infected birds and vaccination of poultry (1). In addition to the economic impact, H5N1 has a high mortality rate in humans coupled with an ever-present threat of pandemic influenza (2). HPAI H5N6 virus (hereafter H5N6) emerged in Vietnam in April 2014 (3, 4).

Both virus subtypes are highly pathogenic in chickens, may cause asymptomatic infection in ducks, and have been associated with sporadic human infection and deaths in Asia. Numerous studies have explored the epidemiology of H5N1 in Asia, describing its spatial distribution at the regional level and in individual countries. Multiple factors have been associated with H5N1 including increased density of domestic ducks (5, 6) and chickens (7), proximity to high aggregations of human population density, a greater percentage of land used as rice paddy fields, higher rice-cropping intensity and lower average annual precipitation (8, 9). These studies predominantly analysed passive surveillance disease presence data resulting in exposure to temporal and spatial variations in surveillance effectiveness. However, contrast to the extensive literature surrounding H5N1, little has been published on the epidemiology of H5N6 in poultry.

In response to the endemic HPAI status in Vietnam, extensive active surveillance of live bird markets (LBMs) was initiated in 2008, first targeting the H5N1 virus and later expanding to include the H5N6 strain. LBMs were chosen as the foci for surveillance activities, partly because funding constraints precluded active surveillance at the farm level, but also because LBMs act as potential reservoirs for HPAI due to their role as hubs for poultry trade (10–12). Moreover, LBMs in northern Vietnam have been found to be highly connected through contact networks, enabling spread of HPAI not only between markets, but also between regions and even across international borders (12, 13). The key role that LBMs play in endemic spread of the virus was highlighted by the impact of the introduction of various biosecurity measures and infection control policies. Requirements such as the introduction of a day of market closure, cleaning at regular intervals, and for all birds to be sold or slaughtered by the end of trading each day greatly reduced the prevalence of HPAI in Hong Kong LBMs (14).

The aim of this study was to analyse the spatial distribution of H5N1 and H5N6 in Vietnamese LBMs using the same set of predictor variables. The majority of studies investigating HPAI in Asia utilise passive surveillance data, which relies upon detection and testing of clinically affected birds. Whilst HPAI H5N1 has been detected in asymptomatic ducks and poultry in LBM (15, 16), such cases are not detected through passive surveillance. The data collected through active surveillance of Vietnamese LBMs over a 5-year period provide a unique opportunity to explore HPAI epidemiology in Vietnam using virus detection data that are less exposed to reporting bias compared with data from passive surveillance. Specific objectives of this study were to (i) determine the prevalence of H5N1 and H5N6 virus in Vietnamese LBMs between 2011 and 2015; (ii) explore the spatiotemporal distributions of H5N1 and H5N6 virus in Vietnam; and (iii) develop models to quantify the spatiotemporal association between agroecological factors and the two HPAI strains using the same set of predictor variables.

## Materials and Methods

### Surveillance Characteristics

#### Surveillance Protocol

Sampling was conducted as part of routine governmental active surveillance. All surveillance activities and protocols were approved by the Vietnamese Department of Animal Health (DAH) Epidemiology Division before implementation.

Sampling activities were implemented at specified times and places by the provincial Sub-Department of Animal Health (SDAH). At the LBM level, a sample size of 30 was required for 95% confidence of detection of H5N1 or H5N6, assuming prevalence of 10%, test sensitivity of 90%, and specificity of 99%. Sample testing was conducted in seven Regional Animal Health Office BSL2+ certified laboratories belonging to DAH, using BSL3 biosafety practice.

The surveillance period extended from September 2011 to December 2015 and was divided into six “Rounds” (Table 1).

**Table 1:** Graphical illustration of the temporal distribution of the six rounds of active surveillance sampling between September 2011 and December 2015.

	January	February	March	April	May	June	July	August	September	October	November	December
2011									Round 1			
2012	Round 1 (cont)									Round 2		
2013	Round 2 (cont)									Round 3		
2014	Round 3 (cont)				Round 4						Round 5	
2015							Round 6					

### LBM Selection

Selection of sampling locations varied by Round as follows:

1. *Round 1*: samples were collected from the two largest LBMs in each of 30 provinces out of a total of 63 (58 provinces and 5 centrally controlled municipalities (cities) at the same level as provinces). Provinces were selected on the basis of fulfilling one or more of the following criteria: (i) having a previous history of HPAI outbreaks, (ii) presence of an international border, (iii) having a high density of poultry, or (iv) high human population density.
2. *Rounds 2–5*: samples were collected from provinces distributed throughout Vietnam using the same criteria for province selection as in Round 1. For every round, the DAH selected (i) 40 provinces from which one small-scale LBM was sampled and (ii) 20 provinces from which a large-scale LBM was sampled. The DAH selected 120 districts from the aforementioned 40 provinces (three districts per province) and 20 cities from the aforementioned 20 provinces.

Districts were selected on the basis of (i) having a high duck density and (ii) having a history of H5N1 outbreaks. In each selected district or city, the SDAH of the corresponding province selected one LBM for sampling. LBMs were selected on the basis of (i) size (at least six vendors), (ii) source of birds (within the district for small-scale LBMs, outside the province for large-scale LBMs), and (iii) no inclusion in H7N9 surveillance activities.

Small-scale markets were defined as markets which draw birds from within the same district and/or province. Sampling should therefore capture/represent the local circulation of HPAI. Large-scale markets were defined as markets which draw birds from outside the province and therefore capture/represent the national and/or regional circulation of HPAI.

3. *Round 6*: DAH selected 32 provinces distributed throughout the country; 12 northern provinces that share a border with China or have poultry trading connected to northern border provinces, and 20 central/southern provinces. In the northern provinces, a total of 48 LBM (4 from each province) were sampled. In the central/southern provinces, the largest LBM in each province was sampled.

### Data Collection

#### Sampling

On the day of sampling, the vendors to be sampled were selected randomly from all vendors present at the market selling more than five ducks (or chickens for Round 6). The number and type of samples collected for each market day according to the surveillance design is summarised in Table 2.

**Table 2:** Target number of samples to be collected per market day, according to round and sample type.

Sample type		Numbers refer to pooled samples when not indicated otherwise			
		Round 1	Rounds 2–4	Round 5	Round 6
Oropharyngeal swabs	Ducks	4	6	6	6
	Chicken	0	0	0	6

Environmental swabs from four large live bird markets	Faeces from cage	0	4 individual samples	0	0
	Waste from resting area	0	4 individual samples	0	0
	Feathers	0	4 individual samples	0	0
	Dirt in slaughter area	0	4 individual samples	0	0

Environmental swabs from all sampling sites	Liquid waste	0	0	2	2
	Solid waste	0	0	2	2
	Faeces	0	0	1	1
	Drinking water	0	0	1	1

*Pooled samples are the combination of 5 swab samples*.

Oropharyngeal swabs from ducks were collected consistently during each surveillance Round, whilst oropharyngeal swabs from chickens were collected during the last Round only. Environmental sampling started during Round 2 with the collection of faeces, feathers and waste in the resting and slaughter areas at four selected large LBMs. Four samples of each type were collected and tested individually. From Round 5 onwards, environmental sampling was extended to all LBMs regardless of their size and environmental swabs were pooled instead of being tested individually. Pooled samples comprised five merged swab samples (either oropharyngeal or environmental). Depending on the Round, between 93 and 100% of the market days reached the sample size targets (detailed in Table 2) for each type of sample.

#### Case Definition

Samples were tested at Regional Animal Health Offices for the H5 and N1 virus subtypes using real-time reverse transcription polymerase chain reaction. From Round 4 onwards samples were also tested for the N6 subtype. Cycle threshold values of less than 35 were regarded as positive. Samples positive for both the H5 and N1 subtypes were classified as positive for H5N1. Similarly, samples positive for both the H5 and N6 subtypes were classified as positive for H5N6.

The epidemiological unit for this study was a market day at a given LBM on a given date. A market day was classified as positive for H5N1 or H5N6 if one or more samples (individual or pooled) collected from that LBM on that date tested positive.

#### Agroecological Predictor Data

A review of the published literature served to identify potential predictor variables previously shown to be risk factors for HPAI occurrence and the final set of predictor variables used in this study included the following: density of ducks (heads/km^2^) (7, 8, 17, 18), density of chickens (heads/km^2^) (7, 8, 18, 19), human population density (heads/km^2^) (6–8, 18, 20), travel time (minutes) to the nearest city with a population of ≥50,000, suitability of areas for growing rice (8, 18), river density (km length/km^2^) (7, 19, 21, 22), average annual precipitation (mm) (23), average monthly minimum temperature (°C), and average monthly maximum temperature (°C) (23–25). LBM density (number of LBM/10 km^2^) was also included.

Digital spatial data layers representing each predictor variable were sourced for Vietnam from the public domain, and all spatial data manipulations and map creation were performed using ArcGIS 10.3.1 (28). Chicken and duck densities were extracted from the *Gridded Livestock of the World* (resolution: 1 km^2^),^1^ and human population density was obtained from *Gridded Population of the World v4* (resolution: 1 km^2^; estimated for 2015).^2^ The predicted density of LBMs/10 km^2^ was obtained from a model generated by Gilbert et al. (unpublished, model description in Supplementary Material; resolution: 10 km^2^), which was resampled to a resolution of 1 km^2^. Travel time to the nearest city was obtained from the Global Environment Monitoring Unit in the Joint Research Centre of the European Commission (26). Areas suitable for rice growing were extracted from *Suitability for Rain-fed and Irrigated Rice (High Input)*, a shapefile available from Food and Agricultural Organization’s GeoNetwork website (published 2007).^3^ The data were converted to raster format (resolution: 1 km^2^), and the original eight suitability categories were recategorised as follows: high (very high/high/good), moderate (medium/moderate), low (marginal/very marginal), unsuitable. Open water features were extracted from *VMap0 Perennial Water Courses (Rivers) of the World* (published 1997) (available from the Food and Agricultural Organization’s GeoNetwork website; see text footnote 3) and density of rivers per square kilometre calculated using the line density feature in ArcGIS. Average monthly precipitation and minimum and maximum temperature data (based on the time frame 1950–2000) were obtained from the WorldClim website ((27); accessed March 2017). A vector shapefile of Vietnam’s provincial boundaries was obtained from the GADM Database of Global Administrative Areas v2.8.^4^ All data were processed to ensure that projections and extents matched. Latitude and longitude were available for LBMs, and data for each predictor variable were extracted to the point location of individual LBMs.

### Statistical Analysis

#### Data Management

The Regional Animal Health Offices used Microsoft Excel to compile the sample collection spreadsheet and laboratory results into a single dataset (regional dataset). These regional datasets were submitted to the DAH each month where they were aggregated to provide a single dataset for each surveillance period. However, merged datasets were not available for some periods, and data recording was not harmonised between regional datasets resulting in multiple names identifying the same location. In such instances, markets with different names but the same coordinates were considered to be the same market. Eighty-three LBMs with missing longitude and latitude data were assigned the coordinates of the relevant commune centroid.

#### Mapping Spatiotemporal Distribution

To preserve LBM anonymity, market days were aggregated by province, and province-level prevalence of H5N1 and H5N6 was calculated for each Round as the number of positive market days in a province divided by the total number of market days sampled in that province.

Choropleth maps of raw rates or standard mortality ratios per area can be misleading; the addition of a small number of cases in an area with a small population at risk can dramatically increase the reported rate of disease for the area. Conversely, the addition of the same number of cases in an area with a large population at risk has little effect on the reported rate of disease for the area. Bayesian approaches allow disease rates to be adjusted through combining the observed rate for an area with rates observed in surrounding areas. When the at risk population of an area of interest is large, and the statistical error of the rate estimate small, higher credibility is given to the observed estimate, and the Bayes adjusted rates are similar to observed rates. However, where the population at risk is small, the rate is adjusted towards the mean rate observed over the wider study area.

Choropleth maps of empirical Bayes-smoothed prevalence were generated for H5N1 and H5N6 using Eqs 1–3 as follows: given that *y_i_* equalled the number of positive market days observed in the *i*th province, *n_i_* the total number of market days sampled in the *i*th province, and *r_i_* was the proportion of positive market days for the *i*th province, then the pooled mean of observed prevalence across all provinces (γ) was calculated as follows:

(1) $$\gamma =\sum \frac{y_{i}}{n_{i}},$$

and the estimate of the population variance of the prevalence based on a weighted sample of the observed prevalences (φ) was calculated as follows:

(2) $$\varphi ={\frac{\sum n_{i}\left(r_{i}-\gamma \right)}{\sum n_{i}}}^{2}-\frac{\gamma }{\bar{n}},$$

then θ, the empirical Bayes-smoothed prevalence for the *i*th province, was calculated as follows:

(3) $$\theta =\frac{\varphi *\left(r_{i}-\gamma \right)}{\varphi +\frac{\gamma }{n_{i}}}+\gamma .$$

#### Exploring Spatial Autocorrelation and Clustering

Spatial autocorrelation of the smoothed Bayes risk was explored at a global scale using the Moran’s *I* statistic and at a local scale using the Anselin Local Moran’s *I* statistic and Getis-Ord GI* statistic. The global Moran’s *I* statistic was used to assess the presence, strength and direction of spatial autocorrelation over the whole study area, using a queen’s contiguity weights matrix and 499 random permutations. A *p*-value ≤ 0.05 was considered significant. The Local Moran’s *I* and GI* statistics were used to detect clustering of provinces with similar risk of H5N1 or H5N6, and to identify the locations of province-level hot and/or cold spots. The GI* statistic returned a *z*-score for each province and for statistically significant positive *z*-scores, the larger the *z*-score the more intense the clustering of high values (hot spot). For statistically significant negative *z*-scores, the smaller the *z*-score the more intense the clustering of low values (cold spot). All spatial analyses were conducted using tools provided in ArcGIS 10.3.1 (28).

#### Modelling Associations Between Agroecological Factors and HPAI

Multivariable logistic regression analyses were used to investigate associations between putative predictor variables and H5N1 or H5N6-positive market days. Univariable analyses for each predictor variable were conducted, with significant variables included in multivariable analysis. All univariable and multivariable statistical analyses were performed in R 3.4.0 (29). Before multivariable analysis, all predictor data were standardised to a mean of 0 and SD of 1, for variables measured at different scales to contribute equally to the analysis. To identify the set of predictors associated with H5N1 and H5N6-positive market days, non-spatial generalised linear models were used, implemented *via* the R *glmulti* package (30), to build every possible non-redundant model for every combination of predictor main effects (interactions were not included due to the number of variables involved). Final best-fit models were chosen using Akaike’s information criterion (AIC), which ranks models based on goodness-of-fit and complexity, whilst penalising deviance. The predictors identified in this first step were then included in a mixed-effects logistic regression model with the variable “market” as a random effect to determine whether any predictors were no longer significant after accounting for non-independence of market days. All continuous variables were assessed for linear trend by comparing the model with the continuous version of the variable with a model where the variable was categorised into quartiles. If the likelihood ratio test *p*-value was <0.05 the categorical version of the variable was included in model.

All identified predictors which remained significant at the 5% level in the mixed-effects logistic regressions were then included in a conditional autoregressive model (CAR) to account for the spatial autocorrelation of observations. Clustering of markets within provinces was accounted for by the inclusion of a spatially varying random effect “Province,” using a spatial weights matrix where polygons were classified as neighbouring if they shared a corner or border (queen’s contiguity). Clustering of market days within markets was accounted for through the inclusion of the non-spatially varying random effect “Market.” The potential temporal effect of sampling heterogeneity was accounted for through inclusion of the variable “Round” in the model.

All CAR models were implemented in R using integrated nested Laplace approximations (INLA) which uses an approximation for inference and avoids the computational demands, convergence issues and mixing problems sometimes encountered by Markov chain Monte Carlo algorithms (31). The model was fitted using R-INLA, with the Besag model for spatial effects specified inside the function. In the Besag model, Gaussian Markov random fields are used as priors to model spatial dependency structures and unobserved effects. In addition, each model was run through INLA whilst excluding the random spatial effect to obtain non-spatial Bayesian estimates and to compare model fit and performance due to the explicit spatial process. Model selection was based on the deviance information criterion (DIC) where a lower DIC indicates a better model fit. In all analyses, an α-level of 0.05 was adopted to indicate statistical significance.

Choropleth maps showing the spatial distribution of the posterior means of the structured random effects obtained from the models were produced in ArcGIS (28).

## Results

### Sampling Sites and Samples

During the surveillance period 22,185 pooled samples were collected from 459 LBM distributed between 48 provinces (242 districts) (Table 3). Each LBM was visited between 1 and 28 times (median 4 visits), providing a total of 3,461 market days for analysis. Sampling intensity was highest in Round 1 and decreased thereafter (Table 3).

**Table 3:** Sampling characteristics and prevalence of highly pathogenic avian influenza H5N1- and H5N6-positive market days of the six surveillance Rounds.

	Rounds						
	1	2	3	4	5	6	Total

Dates	September 2011–February 2012	October 2012–September 2013	October 2013–April 2014	April 2014–October 2014	November 2014–December 2014	July 2015–December 2015	September 2011–December 2015
Number of pooled samples	3,952	4,642	3,984	5,301	1,668	2,638	22,185
Number of provinces	30	44	42	44	44	30	48
Number of districts	122	141	135	138	71	58	242
Number of live bird markets	279	152	143	143	77	63	459
Number of days	153	365	212	184	61	183	1,158
Total market days	974	748	624	827	142	146	3,461
Sampling intensity (market days/number of days)	6.4	2.1	3.0	4.5	2.3	0.8	3.0
Observed prevalence H5N1-positive market days (%)	8.5 (41/974)	19.5 (146/748)	15.7 (111/624)	6.8 (56/827)	14.5 (18/142)	10.2 (15/146)	11.2 (387/3,461)
Observed prevalence H5N6-positive market days (%)				0.7 (6/827)	16.2 (23/142)	26.0 (38/146)	6.01 (67/1,115)

In general, sampled provinces were evenly distributed throughout the country although sampling in Rounds 2–4 provided more homogenous coverage of Vietnam than Rounds 1 and 6, with the latter exhibiting a slight north–south emphasis.

### Prevalence of H5N1 and H5N6-Positive Market Days

The observed prevalence of H5N1-positive market days varied between rounds (median: 12.35; range: 6.8–19.5%) although this difference was not significant (χ^2^ *p*-value = 0.48) (Table 3). The observed prevalence of H5N6-positive market days increased significantly (χ^2^ *p* < 0.001) over Rounds 4–6 from 0.7 to 26% (Table 3).

### Spatiotemporal Distribution of H5N1- and H5N6-Positive Market Days

**Figure 1:**
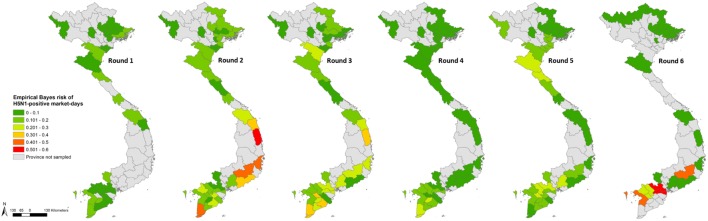
Province-level Bayes risk of highly pathogenic avian influenza H5N1 in Vietnam (Rounds 1–6).

**Figure 2:**
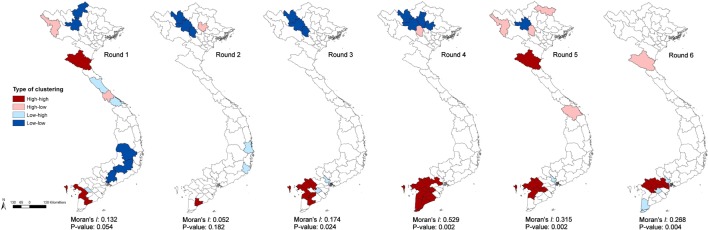
Local Indicators of Spatial Association cluster maps and Moran’s *I* statistics of highly pathogenic avian influenza H5N1 Bayes risk for Rounds 1–6.

**Figure 3:**
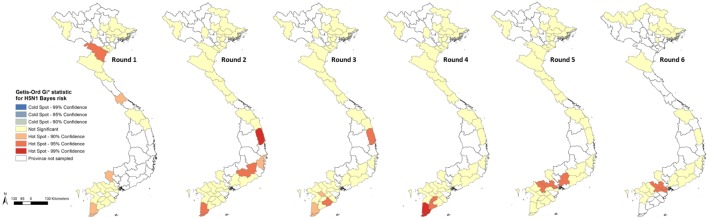
Getis-Ord GI* statistic maps showing hot-spot provinces for highly pathogenic avian influenza H5N1 Bayes risk for Rounds 1–6.

**Figure 4:**
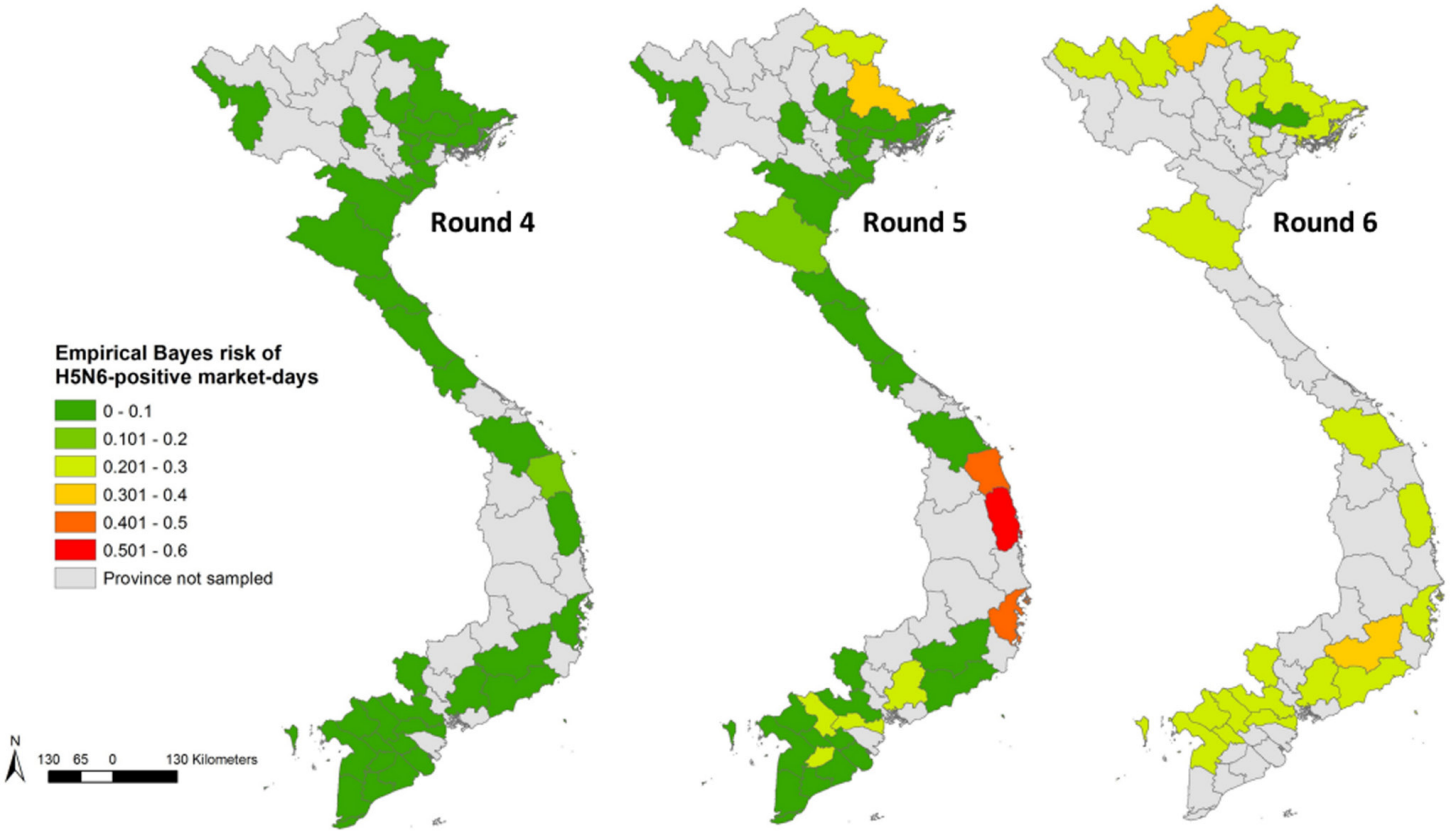
Province-level empirical Bayes risk of highly pathogenic avian influenza H5N6 in Vietnam (Rounds 4–6).

**Figure 5:**
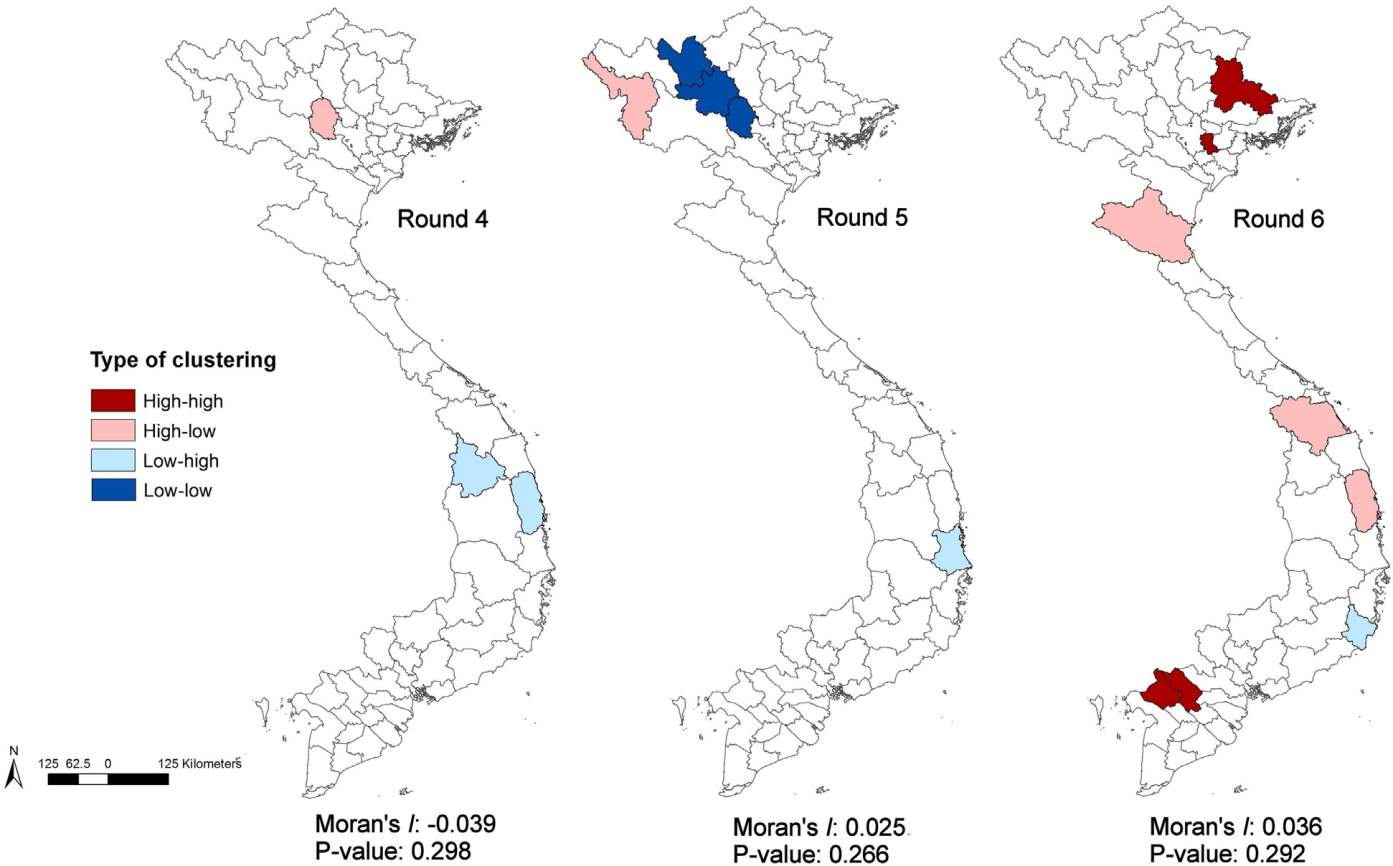
Local Indicators of Spatial Association cluster maps and Moran’s *I* statistics of empirical Bayes risk estimates of highly pathogenic avian influenza H5N6 for Rounds 4–6.

**Figure 6:**
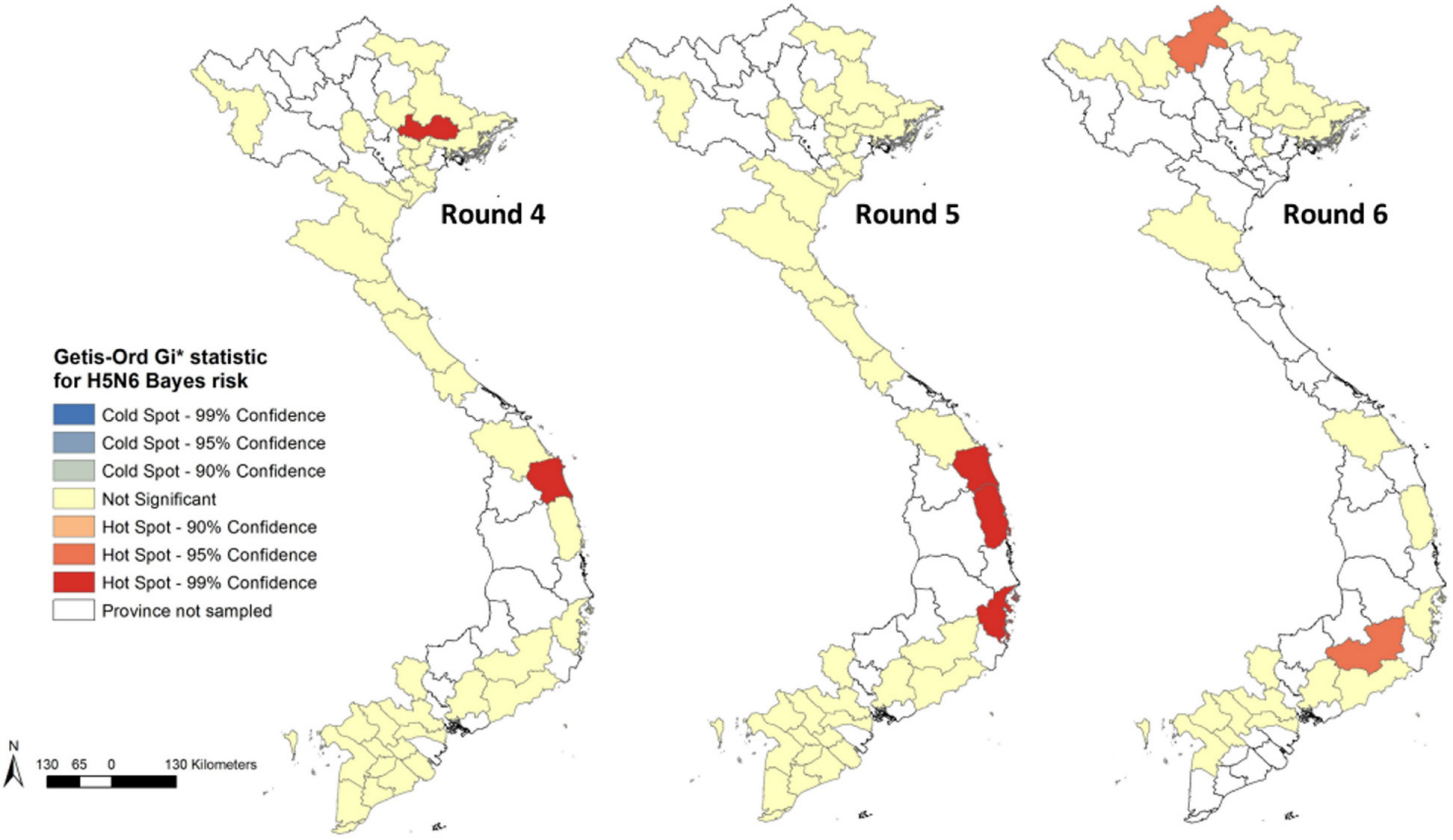
Getis-Ord GI* statistic maps showing hot-spot provinces for highly pathogenic avian influenza H5N6 empirical Bayes risk estimates for Rounds 4–6.

Province-level Bayes-smoothed prevalence of H5N1-positive market days was spatially heterogeneous in all six Rounds, although it was highest in Rounds 2 and 6 (Figure 1). This apparent spatial heterogeneity was supported by both the global and local autocorrelation statistics, which identified significant positive spatial autocorrelation in all rounds except Round 2 (Moran’s *I p*-value > 0.05; Figure 2). The positive autocorrelation was characterised by repeated occurrence, in the south of the country, of a cluster of high-risk provinces surrounded by other high-risk provinces, although the size of the cluster varied between Rounds (Figure 2). Conversely, northern Vietnam was characterised by low-risk provinces surrounded by other provinces of low risk. However, the north also exhibited periodic recurrent outliers; provinces with a high disease risk but surrounded by others with a low disease risk (Figure 2). Hot-spot provinces were identified in all Rounds but the number decreased over time (Rounds 1–3: *n* = 4; Rounds 4–5: *n* = 2; Round 6: *n* = 1) (Figure 3). One province, in particular, Ca Mau was identified as a hot-spot province of H5N1-positive market days in four of the six rounds. Unlike H5N1, province-level empirical Bayes-smoothed risk of H5N6-positive market days did not display any significant spatial heterogeneity in any of the Rounds (Moran’s *I p*-value > 0.05) although the level of risk increased between Rounds 4 and 6 (Figure 4). In general, the most common pattern was one of outliers; provinces with a high risk of H5N6-positive market days were generally surrounded by low-risk provinces and *vice versa* (Figure 5). Two hot-spot provinces were identified in each Round although Quang Ngai was the only province to be identified as a hot spot more than once (Rounds 4 and 5; Figure 6).

### Risk Factors for H5N1 and H5N6-Positive Market Days

The most robust H5N1 multivariable model, based on the AIC, included six of the thirteen predictor variables; suitability for rice-growing, sampling month, average monthly maximum temperature, river density, travel time to a city and chicken density, and were therefore included in the H5N1 INLA model. The variable “Round” was forced into the model to account for temporal variation in sampling. The CAR model based on these variables had a DIC value of 1,984.31 (H5N1). Inclusion of the spatial random effect “province” improved the fit of the H5N1 model by 8.17%, reducing the DIC to, 1,822.10. Three of the six variables retained in the model were statistically significant, three variables were not deemed significant, due to the odds ratio (OR) 95% credible interval crossing 1. The odds of a market day being positive for H5N1 varied between Rounds. Comparison of OR across months identified the likelihood of market day status being positive to be highest in January and February. The odds of a market day being positive for H5N1 were 3.36 (95% CrI 1.29, 8.36) greater where the average maximum temperature was ≥30.33°C compared with areas where the average maximum temperature was ≤24.47°C (Table 4).

**Table 4:** Posterior mean coefficients, odds ratios (ORs), and 95% credible intervals (CrI) of spatial and non-spatial conditional autoregressive models of market days positive for highly pathogenic avian influenza H5N1 virus (Vietnam, 2011–2015).

	Coefficient, posterior mean (95% CrI)		OR, posterior mean (95% CrI)	
	Multivariable model (no spatially varying random effect)	Multivariable model (province as spatially varying random effect)	Multivariable model (no spatially varying random effect)	Multivariable model (province as spatially varying random effect)
Suitability for rice growing				
High/moderate	Baseline	Baseline	Baseline	Baseline
Marginal/unsuitable	0.47 (0.15, 0.78)	−0.04 (−0.51, 0.42)	1.60 (1.17, 2.19)	0.96 (0.60, 1.52)

Sampling month				
January	Baseline	Baseline	Baseline	Baseline
February	−0.27 (−0.67, 0.12)	−0.36 (−0.77, 0.05)	0.76 (0.51, 1.13)	0.70 (0.46, 1.05)
March	−0.87 (−1.40, −0.36)	−1.00 (−1.55, −0.47)	0.42 (0.25, 0.70)	0.37 (0.21, 0.63)
April	−1.01 (−1.55, −0.49)	−1.11 (−1.68, −0.57)	0.37 (0.21, 0.62)	0.33 (0.19, 0.57)
May	−1.48 (−2.19, −0.82)	−1.75 (−2.49, −1.06)	0.23 (0.11, 0.44)	0.17 (0.08, 0.35)
June	−0.27 (−1.19, 0.60)	−0.59 (−1.51, 0.29)	0.76 (0.30, 1.83)	0.55 (0.22, 1.34)
July	−0.50 (−1.47, 0.43)	−0.82 (−1.79, 0.12)	0.61 (0.23, 1.54)	0.44 (0.17, 1.13)
August	−0.35 (−1.26, 0.53)	−0.65 (−1.58, 0.26)	0.70 (0.20, 1.70)	0.52 (0.21, 1.30)
September	−0.80 (−1.60, −0.05)	−1.10 (−1.92, −0.33)	0.45 (0.20, 0.95)	0.33 (0.15, 0.72)
October	−1.80 (−3.44, −0.49)	−1.96 (−3.62, −0.62)	0.16 (0.03, 0.61)	0.14 (0.03, 0.54)
November	−0.53 (−0.99, −0.08)	−0.66 (−1.13, −0.20)	0.59 (0.37, 0.92)	0.52 (0.32, 0.82)
December	−0.80 (−1.20, −0.40)	−0.92 (−1.34, −0.51)	0.45 (0.30, 0.67)	0.40 (0.26, 0.60)

Average maximum temperature (°C)				
≤24.47	Baseline	Baseline	Baseline	Baseline
24.48–28.74	0.72 (0.26, 1.20)	0.15 (−0.75, 1.03)	2.06 (1.29, 3.34)	1.17 (0.47, 2.81)
28.75–30.32	1.71 (1.29, 2.15)	1.12 (0.21, 1.99)	5.54 (3.63, 8.62)	3.07 (1.23, 7.32)
≥30.33	1.69 (1.23, 2.16)	1.21 (0.26, 2.12)	5.41 (3.43, 8.71)	3.36 (1.29, 8.36)

River density (km length/km^2^)	0.08 (−0.05, 0.21)	−0.05 (−0.29, 0.19)	1.08 (0.95, 1.23)	0.95 (0.75, 1.21)

Travel time (min) to nearest city with population ≥50,000	−0.18 (−0.33, −0.04)	−0.07 (−0.24, 0.08)	0.84 (0.72, 0.96)	0.93 (0.78, 1.09)

Chicken density (heads/km^2^)				
<285	Baseline	Baseline	Baseline	Baseline
285–791.3	0.19 (−0.13, 0.51)	0.03 (−0.39, 0.45)	1.21 (0.88, 1.66)	1.03 (0.68, 1.57)
791.4–1,686.1	0.07 (−0.27, 0.41)	0.00 (−0.41, 0.42)	1.07 (0.77, 1.50)	1.00 (0.66, 1.52)
≥1,686.2	−0.44 (−0.86, −0.03)	−0.37 (−0.87, 0.12)	0.64 (0.42, 0.97)	0.69 (0.42, 1.13)

Round				
Round 1	Baseline	Baseline	Baseline	Baseline
Round 2	1.11 (0.76, 1.46)	1.14 (0.76, 1.52)	3.02 (2.13, 4.31)	3.13 (2.14, 4.57)
Round 3	0.85 (0.48, 1.21)	0.85 (0.47, 1.24)	2.33 (1.62, 3.35)	2.35 (1.60, 3.56)
Round 4	−0.12 (−0.85, 0.61)	0.06 (−0.68, 0.81)	0.89 (0.43, 1.85)	1.06 (0.51, 2.25)
Round 5	0.68 (−0.02, 1.33)	0.76 (0.03, 1.44)	1.97 (0.98, 3.78)	2.14 (1.03, 4.23)
Round 6	1.25 (0.18, 2.29)	1.57 (0.47, 2.65)	3.48 (1.19, 9.91)	4.81 (1.60, 14.16)

Model deviance information criterion	1,934.81	1,822.10		

*Province included as a spatially varying random effect*.

In the multivariable analysis, only three of the thirteen predictors were significantly associated with market days being positive for H5N6: river density, human population density and market density. These covariates were taken forward to the H5N6 INLA model. The variable “Round” was forced into the model to account for temporal variation in the sampling. The CAR model based on these variables had a DIC value of 202.36, inclusion of the spatial random effect “province” did not improve the model fit (the DIC was lowered by 0.03% to 202.29). Therefore, the final model used for H5N6 therefore did not include the spatially varying random effect. The likelihood of a market day being positive for H5N6 was higher with successive Rounds and lower river density (Table 5).

**Table 5:** Posterior mean coefficients, odds ratios (ORs), and 95% credible intervals (CrI) of non-spatial conditional autoregressive models of market days positive for highly pathogenic avian influenza H5N6 virus (Vietnam, 2011–2015).

	Coefficient, posterior mean (95% CrI)Multivariable model (no spatially varying random effect)	OR, posterior mean (95% CrI)Multivariable model (no spatially varying random effect)
River density (km length/km^2^)	−0.74 (−1.17, −0.34)	0.48 (0.31, 0.71)

Human population density (heads/km^2^)	0.28 (−0.08, 0.61)	1.31 (0.92, 1.84)

Market density (live bird market/10 km^2^)					
≤2.8	Baseline	Baseline
2.81–4.64	−0.15 (−1.45, 1.16)	0.86 (0.24, 3.19)
4.65–8.91	0.90 (−0.24 2.14)	2.47 (0.78, 8.52)
≥8.92	0.28 (−1.01, 1.61)	1.32 (0.36, 4.99)

Round		
Round 4	Baseline	Baseline
Round 5	3.37 (2.39, 4.48)	26.06 (9.84, 78.44)
Round 6	3.72 (2.62, 4.92)	40.48 (13.61, 133.16)

Model deviance information criterion	200.94	

**Figure 7:**
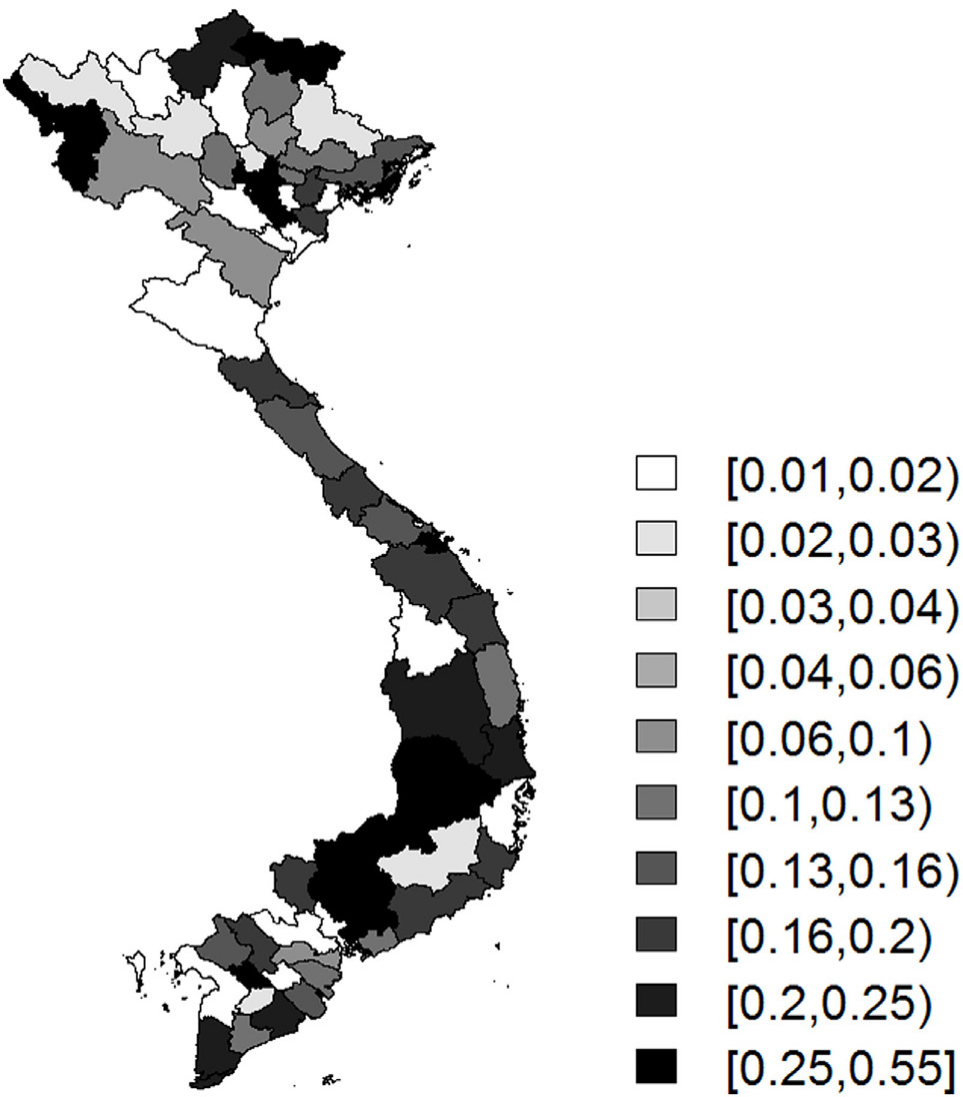
Choropleth map showing the province-level posterior mean probabilities of the spatially structured random effect for H5N1.

Mapping posterior means of the spatially structured random effects for H5N1 showed them to be reasonably homogenously distributed throughout the country, suggesting that there is unexplained variation in most regions, after accounting for the model covariates (Figure 7).

## Discussion

The results of this study suggest that the epidemiology of H5N1 and H5N6 in Vietnam are very different. Not only do the two strains show different distributions, they are also associated with different risk factors. Whilst the risk of H5N6-positive market days was homogenous across Vietnam, the posterior mean probabilities of H5N1 from the CAR model at the province level showed clear regional differences, with higher probabilities in the southern and central provinces of Vietnam. Similarly, whilst higher risk of H5N6-positive LBMs was associated with lower river density, spatial variation in H5N1 risk was primarily associated with climatic factors.

Collection month was associated with variation in market-day H5N1 risk. The odds of H5N1-positive market days were highest in January and February. No samples collected between January and March were tested for H5N6. Seasonal fluctuations in the proportion of positive market days may be due to a combination of climatic factors and peaks in demand for poultry products. Higher incidence of H5N1 in domestic poultry in central and southern Vietnam has been shown to coincide with an increased demand for poultry products in January and February associated with the Lunar New Year Festival (32). Colder temperatures in winter months have also been proposed to contribute to higher risk of H5N1 due to longer virus survival time in the environment (24, 25).

Higher average maximum temperature was associated with higher risk of market day H5N1 positivity. This factor contributes to the observed north/south risk divide, as average maximum temperatures are higher in southern than northern provinces of Vietnam. However, due to limitations in sampling strategy, with time gaps in surveillance and variation in sampling strategy between years, it is not possible to reliably assess consistency of seasonal patterns over time.

Rice-cropping intensity has previously been associated with H5N1 presence in South East Asia (18). None of the samples collected on the 28 market days in areas with poor suitability for rice production tested positive for H5N1. Similarly, of the six market days sampled for H5N6 in areas with poor suitability for rice production, no samples tested positive. However, a significant association between the risk of H5N1-positive market days and the higher suitability of land for growing rice was not identified in this study, which may be due to the small number of market days sampled in poor rice production areas. Remote sensing data can capture greater resolution compared with traditional census collection, allowing for greater accuracy in assessment of rice-cropping intensity and suitability. This finer scale resolution may improve detection of associations between rice growing and prevalence of HPAI (18).

The purpose of inclusion of variables such as chicken density, duck density, and rice suitability was to capture risk factors at point of production. Consideration must be given to the potential for chicken and duck farms to be located in geographically distant areas from the market (33). Contrary to findings of some previous studies in South East Asia, higher domestic chicken population density and waterfowl density were not found to be associated with increased risk of H5N1-positive market days (8, 17). The production location of poultry from which samples were collected was not obtained during the study. Data used in the models are reflective of the locality of the market, but not necessarily the location of production. Therefore, caution is necessary regarding the interpretation of the association between risk of a market being positive for H5N1 or H5N6 and variables relating to location of production including chicken density, duck density, and the suitability of land for rice production. The prevalence of HPAI at the LBM level will be impacted by the catchment area and the extent of interconnectedness with other LBM through poultry trade. Identification of production location would enable capture of risk associated with production factors with greater accuracy.

Reduced travel time to a major city has been associated with higher risk of H7N9 presence in LBM in Asia (34). Travel time to the nearest city is a measure of accessibility of the LBM, and the increased risk associated with more accessible LBM could be reflective of birds being drawn from more diverse populations, over a larger catchment area and connections with other LBM. In the multivariable INLA model, shorter travel time to the nearest city was not significantly associated with higher H5N1-positive market-day status. This may be due to the highly connected nature of LBM in Vietnam, enabling dissemination even between relatively less well accessed markets (12, 13).

The residual spatial variation in H5N1 market-day risk at the province level indicates that there are unexplained factors contributing to risk that were not included in the model. Vaccination has been used to control HPAI in Vietnam and may have contributed to the spatial and temporal variation in risk, as vaccine coverage has been found to vary with both district and season (35). In addition, the predominant duck breeds may vary between regions and vaccine response of different breeds of domestic ducks to the commercial vaccines has been shown to differ, resulting in shedding continuing in some clinically unaffected, vaccinated ducks (36–38).

Additional market level factors not included in the model have the potential to contribute to between-market variation in the likelihood of a sample testing positive for H5N1 or H5N6. Factors include the number of days per week the market opens, biosecurity measures, length of holding of birds in the LBM, number of birds and stocking density in the LBM, biosecurity, and husbandry on farms producing the poultry for sale (39, 40). Collection of further market level information would enable further improvement of understanding of both H5N1 and H5N6 epidemiology in Vietnam.

Previous studies mapping the spatial distribution of avian influenza in Vietnam have utilised data obtained through passive surveillance (8, 18). In Vietnam, passive surveillance is conducted through farmers or community animal health workers notifying local state vets, with subsequent diagnostic testing and investigation. Positive samples are then reported to the central government. Currently, a low number of outbreaks are reported through passive surveillance (32) due to the reliance on clinical detection of disease, testing, and reporting processes. During this study, samples were collected through active surveillance, with standardised selection criteria across regional areas within each round of sample collection. This approach enables detection of HPAI in subclinical birds and minimises temporal and spatial variation in surveillance effectiveness, enabling more robust identification of regional differences in the prevalence of H5N1 and H5N6 at the level of the LBM. The ongoing active surveillance conducted in LBM is essential to monitor changes in spatiotemporal distribution patterns and strain evolution. Sampling continues to be focussed upon LBM and provinces where prevalence of infection has previously been detected to be highest.

One of the main limitations of the data analysed in this study is that the sampling strategy was not consistent between Rounds. Sampling at the district level was randomised for Round 1, then from Round 2 onwards the sampling strategy at the district level was to target districts with higher risk of H5N1 infection. The variation in odds of a market-day testing positive for H5N1 or H5N6 between Rounds may reflect temporal variation in risk, however, is augmented by the differences in sampling strategies implemented in different rounds. In addition, the sampling strategy at the level of the LBM was not perfectly sensitive; not all birds were sampled and AI positive birds may have been clustered in only part of an LBM. Due to the potential for under-detection, the observed proportion of HPAI positive market days may be lower than the true proportion of HPAI positive market days. In addition, aggregating market days by province, for reasons of anonymity, will have resulted in some loss of within-province heterogeneity. However, as the provinces with the highest risk were also the smallest (southern) provinces, this loss of information is expected to be comparatively small.

In conclusion, this study suggests that the spatial patterns and risk factors are very different for H5N1 and H5N6 in Vietnam. Whilst H5N1 distribution was spatially heterogeneous with significant clustering of high-risk provinces in the south, H5N6 was homogenously distributed. In addition, the likelihood of H5N1 detection at LBM was primarily associated with climatic factors. The different epidemiology of these two HPAI virus strains in Vietnam suggests the need for different surveillance and control approaches.

## Ethics Statement

Data were generated as part of routine governmental active surveillance monitoring of avian influenza in Vietnam, coordinated by the Department of Animal Health, Ministry of Agriculture and Rural Development in Vietnam. All surveillance activities and protocols were approved by the Vietnamese Department of Animal Health (DAH) Epidemiology Division before implementation.

## Author Contributions

PL, SN, KI, and PP contributed to the design of the study. KS, KM, AM, DE, GF, and DP contributed to the data analysis plan. AM organised the database. KS, KM, AM, and DE performed the statistical analyses. KM wrote the first draft of the manuscript. KS and AM wrote sections of the manuscript. KS, KM, AM, GF, DP, TV, and MG contributed to interpretation of results. All the authors contributed to manuscript revision, read and approved the submitted version.

## Disclaimer

The views expressed in this information product are those of the author(s) and do not necessarily reflect the views or policies of FAO.

## Conflict of Interest Statement

The authors declare that the research was conducted in the absence of any commercial or financial relationships that could be construed as a potential conflict of interest.

## Footnotes

^1^https://livestock.geo-wiki.org/home-2/ (Accessed: March, 2017).

^2^http://sedac.ciesin.columbia.edu/data/collection/gpw-v4 (Accessed: March, 2017).

^3^http://www.fao.org/geonetwork/srv/en/main.home (Accessed: March, 2017).

^4^http://www.gadm.org/ (Accessed: March, 2017).

## Abbreviations

HPAI, highly pathogenic avian influenza; LBM, live bird market; INLA, integrated nested Laplace approximations; LISA, local indicators of spatial association; DAH, Department of Animal Health; CAR, conditional autoregressive model.
