# Supplementary material for: Comparative Epidemiology of Highly Pathogenic Avian Influenza Virus H5N1 and H5N6 in Vietnamese Live Bird Markets: Spatiotemporal Patterns of Distribution and Risk Factors
Source: Front Vet Sci. 2018 Apr 5;5:51. doi: 10.3389/fvets.2018.00051 (PMC5896172; doi:10.3389/fvets.2018.00051)
Supplement: Supplementary file 1 [file data_sheet_1.docx]

# Supplementary Material

## Live bird market density model

The LBM model was built based on a census of LBM per commune that was made by the province veterinary officers. The number of LBM was available for 3,399 communes out of a total of 11,382. In each sampled commune, we estimated the LBM density in number of LBM / km^2^ and this density was rasterized at a spatial resolution of 0.083333 decimal degrees (approximately 10 km at the equator). A model was then build using the density from the pixels including surveyed communes using four spatial predictors: i) the human population density from the Worldpop database (Gaughan et al., 2013), ii) the travel time to any city with over 50,000 inhabitants (Nelson, 2008), and iii) the chicken and duck density from the GLW (Robinson et al., 2014). Among a set of possible modelling approaches, the model providing the best prediction was a Boosted Regression Tree (BRT) model (Elith et al. 2008), which provided a correlation of 0.796 between the observed and predicted LBM density per sampled pixel. This model was then reapplied to all pixels of Vietnam as a metric of LBM density, before being resampled at 1 km^2^ resolution to match the scale of the analysis.

### References

Elith, J., Leathwick, J., Hastie, T. (2008) A working guide to boosted regression trees. *J Anim Ecol*, doi: 10.1111/j.1365-2656.2008.01390.

Gaughan, A., Stevens, F., Linard, C., Jia, P., and Tatem, A. (2013). High Resolution Population Distribution Maps for Southeast Asia in 2010 and 2015. *PLoS ONE* 8, e55882. doi:10.1371/journal.pone.0055882.

Nelson, A. (2008) Travel Time to Major Cities: A Global Map of Accessibility. Global Environment Monitoring Unit—Joint Research Centre of the European Commission, Ispra, Italy.

Robinson, T., Wint, G., Conchedda, G., Van Boeckel, T., Ercoli, V., Palamara, E., Cinardi, G., D'Aietti, L., Hay, S., and Gilbert, M. (2014). Mapping the Global Distribution of Livestock. *PLoS ONE* 9, e96084. doi:10.1371/journal.pone.0096084.
